# Mediating Effects of Psychological States on Work Performance of Visiting Nurses According to COVID-19 Workplace Quarantine Measures: A Multi-Group Path Analysis Study

**DOI:** 10.3390/ijerph19010444

**Published:** 2021-12-31

**Authors:** Jee-Hyun Hwang

**Affiliations:** Department of Public Health, Graduate School, The Catholic University of Korea, Seoul 06591, Korea; hjh2830425@catholic.ac.kr

**Keywords:** visiting nurses, experiences of verbal violence, psychological states, difficulties in work performance, workplace quarantine measures

## Abstract

This multi-group path analysis study investigated the effects of verbal abuse (suspicion of infection and disrespect) on difficulties in work performance according to the workplace quarantine measures of visiting nurses during the COVID-19 pandemic. A total of 262 visiting nurses in Korea completed the online questionnaire between 10 October and 30 November 2020, and their data were included in the final analysis. The study found that experiences of verbal abuse mediated fear and anxiety to affect difficulties in work performance. In the path model of the group with a high level of workplace quarantine measures, experiences of verbal abuse (suspicion of infection and disrespect) did not directly affect fear. The opposite was true for the group with low levels of workplace quarantine measures. The implications for the field are that the following is required: heightened awareness of verbal abuse; workplace quarantine policies; and mental health management systems and intervention programs to detect the early fear and anxiety of visiting nurses.

## 1. Introduction

In the past two decades, the world has experienced five public health emergencies: Corona Virus Disease 2019 (COVID-19), Ebola Virus Disease (EVD), Middle East Respiratory Syndrome (MERS), Novel Swine-origin Influenza A (H1N1), and Severe Acute Respiratory Syndrome (SARS). These novel infectious diseases have severely threatened the health of mankind. In particular, COVID-19 and the H1N1 pandemic have had great impacts, not only on healthcare, but also on society and the economy, suggesting an active need for response to pandemics in the future [1].

The first confirmed case of COVID-19 in Korea was diagnosed on 20 January 2020. To date (as of 22 November 2021), a total of 418,252 confirmed cases and 3298 fatalities have been observed, leading to social chaos [2]. Recently, as the social expectations of medical staff have increased due to changes in the health environment, the role of visiting nurses who directly care for patients in the local community has become increasingly important [3,4]. In particular, visiting nurses play an essential role in the local community, promoting public health projects for the comprehensive and continuous health management of the public [3]. Moreover, during pandemics of infectious diseases, challenges for visiting nurses have increased due to the fear of spreading and managing infectious diseases, and the wearing of protective equipment [5]. Therefore, visiting nurses may be more burdened with spreading infectious diseases in local communities than clinical nurses registered in hospitals, and quarantine measures in workplaces may affect the performance of visiting nurses. To improve the safety and health of nurses who visit the homes of patients to provide nursing care during infectious disease pandemics and who are thus exposed to various risks, studies must actively investigate workplace quarantine measures.

During the COVID-19 pandemic, nurses were given heavy workloads in poor environments and experienced psychological distress [6]. Previous studies showed that nurses experienced increased anxiety [7,8,9] and fear of infecting close family and friends [8,10] during pandemics. In particular, unlike registered nurses, visiting nurses have a high risk of experiencing work-related violence as they directly enter patients’ homes for nursing care [11]. Workplace violence includes physical assault, threats of assault, and verbal abuse. In a study on health workers visiting public health centers [12], verbal abuse was the most common form of violence. Verbal abuse can cause fear and negative psychological states such as anxiety [12]. Similarly, intensive care unit (ICU) nurses experience psychological stress such as fear and anxiety following verbal violence [13]. These findings support the hypothesis that experiences of verbal violence cause fear and anxiety. Moreover, psychological stress experiences such as fear and anxiety lead to decreased work motivation and nursing performance [13]. Subsequently, verbal abuse may further decrease nursing quality and productivity [12]. Thus, it is important to assess the mediating effects of fear and anxiety related to the effects of verbal abuse experience on work performance difficulties.

Most studies on verbal abuse, workplace quarantine measures, psychological states (fear or anxiety), and difficulties in work performance during infectious disease pandemics have investigated the interacting effects of these factors [4,6,7,8,9,10,14,15]. However, there is a lack of studies on the relationship between variables according to the level of workplace quarantine measures. A previous study reviewed the level of local government quarantine measures for COVID-19 as a regulatory role in Seoul citizens [16]. However, only a few studies have investigated the relationship between the variables in respect of the level of workplace quarantine measures. Thus, differences according to workplace quarantine measures need to be considered. In particular, studies must assess difficulties in work performance mediated by psychological states (fear and anxiety) as a result of verbal abuse.

Therefore, this study aimed to evaluate the mediating effects of psychological states (fear and anxiety) on difficulties in work performance according to the level of workplace quarantine measures in visiting nurses. Based on the effects of verbal abuse on difficulties in work performance described in previous studies [12,13], multi-group path analysis was conducted to confirm the mediating effects of psychological states (fear and anxiety) on difficulties in work performance in groups with low and high levels of workplace quarantine measures. A hypothetical path model of workplace quarantine measures as a moderating variable was constructed based on a study by Holroyd and McNaught [5] in which low levels of workplace quarantine measures further increased difficulties in work performance.

This study intended to contribute to understanding the difficulties in performing the duties of visiting nurses, solutions to the difficulties, and effective personnel management policies required for safety and health management. In particular, this study is significant in that differences were assessed according to the level of quarantine measures in preparation for potential infectious disease pandemics in the future.

**Figure 1:**
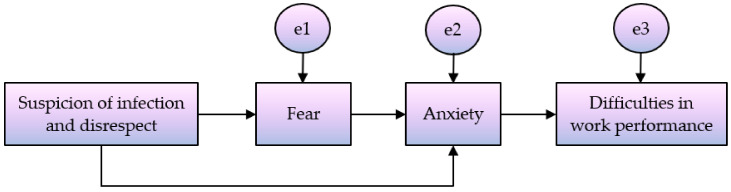
Conceptual framework for this study.

The present study, a hypothetical model was established to explain the difficulties in performing nursing tasks of visiting nurses during the COVID-19 pandemic. The purpose of this study was to investigate the mediating effect of psychological states (fear or anxiety) on the relationship between the experience of verbal abuse (suspicion of infection and disrespect) and difficulties in performing work. Multi-group path analysis was conducted to identify differences according to the level of workplace quarantine measures (Figure 1).

## 2. Materials and Methods

### 2.1. Study Sample

Visiting nurses who understood the purpose of this study and voluntarily agreed to participate were selected through a convenience sampling method. A structured online questionnaire was used to collect data. The questionnaire survey was conducted from 10 October to 30 November 2020. There were no missing values in the questionnaires. A total of 262 visiting nurses completed the online questionnaire and their data were included in the final analysis. Two of the final analysis subjects had been confirmed positive for COVID-19.

### 2.2. Measurements and Variable Definitions

#### 2.2.1. Experiences of Verbal Violence (Suspicion of Infection and Disrespect)

For experiences of verbal abuse (suspicion of infection and disrespect), the number of times the participants experienced verbal abuse or behaviors, such as suspicion of infection and disrespect, during the COVID-19 pandemic was assessed. A higher value indicated more experiences of such violence.

#### 2.2.2. Fear

Fear was assessed with a tool in the COVID-19 risk awareness survey that was used to evaluate fear [17]. Fear related to COVID-19 included a total of eight items: ‘fear of possible confirmed case at work’, ‘fear of being infected’, ‘fear of self-isolation after close contact with an infected person’, ‘fear of possible asymptomatic infection’, ‘fear of disadvantage at work when using sick leave due to infection’, ‘fear of criticism at work for being infected’, ‘fear of others who do not self-report despite suspected symptoms’, and ‘fear of others having suspected or asymptomatic infection’. The items were evaluated on a five-point Likert scale from one point (strongly agree) to five points (strongly disagree), which were reverse-coded. A higher point indicated greater fear for each item. Cronbach’s α of the tool was 0.898.

#### 2.2.3. Anxiety

Anxiety about the risk of COVID-19 infection was evaluated on a numerical rating scale with 10 points for ‘very anxious’ and 0 point for ‘not anxious at all’. A higher value indicated higher anxiety.

#### 2.2.4. Difficulties in Work Performance

Difficulties in work performance was evaluated on a numeral rating scale with 10 points for ‘very much’ and 0 point for ‘not at all’. A higher value indicated greater difficulties in work performance.

#### 2.2.5. Workplace Quarantine Measures

Workplace quarantine measures were evaluated using a total of 13 items: ‘temperature check before entering’, ‘screen dividers in the office’, ‘screen dividers in the cafeteria,’ ‘increased space between desks’, ‘hand sanitizer’, ‘ventilation facilities’, ‘disinfection of public spaces such as office and elevator,’ ‘disinfection of computers and telephones,’ ‘cancellation of business trips and group dinners’, ‘closure of rest rooms and smoking rooms’, ‘shift system for lunch time’, ‘telecommuting and commuting time adjustment’, and ‘leave for workers with respiratory symptoms’. The items were evaluated on a two-point scale of one point (yes) and 0 point (no). A higher score indicated higher levels of quarantine measures in the workplace.

### 2.3. Statistical Analysis

The collected data were analyzed using SPSS 23 and AMOS 20. Cronbach’s α coefficient was measured to verify the reliability of the measurement tools used in the study. Descriptive analysis was conducted for the experience of verbal abuse (suspicion of infection and disrespect), fear, anxiety, difficulty in performing work, and level of workplace quarantine measures. Pearson’s correlation analysis was performed to assess the correlation between the main study variables. Amos 20 was used to verify the goodness-of-fit of the constructed model based on the Tucker–Lewis Index (TLI), Comparative Fit Index (CFI), Root Mean Square Error of Approximation (RMSEA), and Standardized Root Mean Squared Residual (SRMR). To confirm the effect of the relationship between the paths of the model, statistical significance of β and *p* values for each path coefficient was checked. Bootstrap verification was performed to verify the indirect effects. Multi-group analysis was conducted to verify differences in the effect relationship between the variables according to workplace quarantine measures, and Δχ^2^ statistics and *p* values were evaluated to confirm the differences. The participants were divided into groups with high and low levels of workplace quarantine measures, and β and *p* values were assessed to confirm the significance of each path.

### 2.4. Ethical Considerations

This study was conducted after being approved by the institutional review board of the Catholic University of Korea (IRB; MC20QISI0123) and performed in accordance with the Declaration of Helsinki.

## 3. Results

### 3.1. Descriptive Statistics of Measurement Variables

Descriptive statistical analysis was performed for the main variables of the study. The participants experienced verbal violence (suspicion of infection and disrespect) in the range of 0 to 50 times with an average of 1.810; however, skewness and kurtosis were high. Thus, log transformation was performed. Fear scores ranged between 18 and 40 points within a mean score of 33.637 points. Anxiety scores were between one and 10 points with an average of 7.940 points. Difficulty in work performance was measured on a scale of 0 and 10 points with an average of 9.060 points. Workplace quarantine measures scores ranged between one and 13 points, with a mean score of 4.366 points (Table 1).

### 3.2. Correlation between Measurement Variables

Pearson’s correlation analysis was performed to assess the correlation between the main study variables.

Experience of verbal abuse (suspicion of infection and disrespect) was positively correlated with fear (r = 0.181, *p* < 0.010), anxiety (r = 0.197, *p* < 0.010), and difficulties in work performance (r = 0.168, *p* < 0.010). Fear was positively correlated with anxiety (r = 0.495, *p* < 0.010) and difficulties in work performance (r = 0.266, *p* < 0.010). Additionally, anxiety and difficulties in work performance were positively correlated (r = 0.372, *p* < 0.010).

### 3.3. Path Model Goodness-of-Fit

Herein, experiences of verbal abuse primarily mediated fear, and this study aimed to examine the secondary mediating effects of anxiety on difficulties in work performance. The following path model was constructed:

To verify the goodness-of-fit of the constructed model, TLI, CFI, and RMSEA, which are used when the number of samples is large, were assessed [18]. In general, TLI and CFI greater than 0.90 and RMSEA less than 0.08 are considered to indicate good fit of the models [19]. In this study, TLI = 0.925, CFI = 0.975, RMSEA = 0.077 and SRMR = 0.028, suggesting that the model had a good fit.

### 3.4. Significance of Path Coefficients

To investigate the effect relationship between paths of the model, statistical significance was evaluated for each path coefficient.

The path from experience of verbal abuse (suspicion of infection and disrespect) to fear was positively significant (β = 0.181, *p* = 0.003), and the path from experience of verbal violence (suspicion of infection and disrespect) to anxiety was also positively significant (β = 0.111, *p* = 0.041). The path from fear to anxiety was positively significant (β = 0.475, *p* < 0.001), and the path from anxiety to difficulties in work performance was also positively significant (β = 0.372, *p* < 0.001). Altogether, these findings suggested that more experiences of verbal violence (suspicion of infection and disrespect) are associated with greater fear and anxiety, that higher fear leads to higher anxiety, and that higher fear causes greater difficulties in work performance (Table 2).

### 3.5. Evaluation of Indirect Effects

Bootstrap verification was performed to verify the indirect effects. The number of bootstrap samples was 5000, and significance at the 95% confidence level was assessed. The 95% confidence interval for the two indirect effect paths did not include 0, suggesting that the indirect effect was statistically significant.

The experience of verbal violence (suspicion of infection and disrespect) sequentially mediated fear and anxiety to indirectly have significant effects on difficulties in work performance (B = 0.081, *p* = 0.001). In addition, the experience of verbal violence (suspicion of infection and disrespect) mediated anxiety to have significant indirect positive effects on difficulties in work performance (B = 0.104, *p* = 0.017) (Table 3).

### 3.6. Differences in Effect Relationships between Variables According to Workplace Quarantine Measures (Multi-Group Analysis)

The participants were divided into groups with high and low levels of workplace quarantine measures based on the median value. Multi-group analysis was conducted to evaluate differences in the effect relationship between variables according to the level of workplace quarantine measures. A model in which the routes of groups with high and low levels of workplace quarantine measures were equally constrained and another model without any restrictions were compared. As a result, significant differences were observed between the two models (Δχ^2^ = 26.995, *p* = 0.042). This suggested significant differences in the effect relationship between variables according to workplace quarantine measures. Therefore, to assess the differences in the effect relationship between the variables, the participants were divided into groups with high and low levels of workplace quarantine measures, and the significance of each path was evaluated.

In the group with low workplace quarantine measures, verbal abuse (suspicion of infection and disrespect) experience had positive effects on fear (β = 0.198, *p* < 0.011). In contrast, in the group with high workplace quarantine measures, verbal abuse (suspicion of infection and disrespect) experience did not have significant effects on fear. In other words, greater experience of verbal abuse (suspicion of infection and disrespect) was associated with greater fear, only in the group with low workplace quarantine measures. There were no significant differences in paths for the experience of verbal violence (suspicion of infection and disrespect) and fear between the two groups.

In the group with low workplace quarantine measures, verbal abuse (suspicion of infection and disrespect) experience did not have significant effects on anxiety. Similarly, in the group with high workplace quarantine measures, experience of verbal abuse (suspicion of infection and disrespect) did not have significant effects on anxiety. In both groups, experience of verbal abuse (suspicion of infection and disrespect) did not significantly affect anxiety. There were no significant differences in paths for the experience of verbal abuse (suspicion of infection and disrespect) and anxiety between the two groups.

In the group with low workplace quarantine measures, fear had positive effects on anxiety (β = 0.521, *p* < 0.001). Additionally, in the group with high workplace quarantine measures, fear had positive effects on anxiety (β = 0.428, *p* < 0.001). In both groups, greater fear was associated with greater anxiety. There was a significant difference in the path for fear and anxiety between the two groups (Critical Ratios = 2.785 > 1.960).

**Figure 2:**
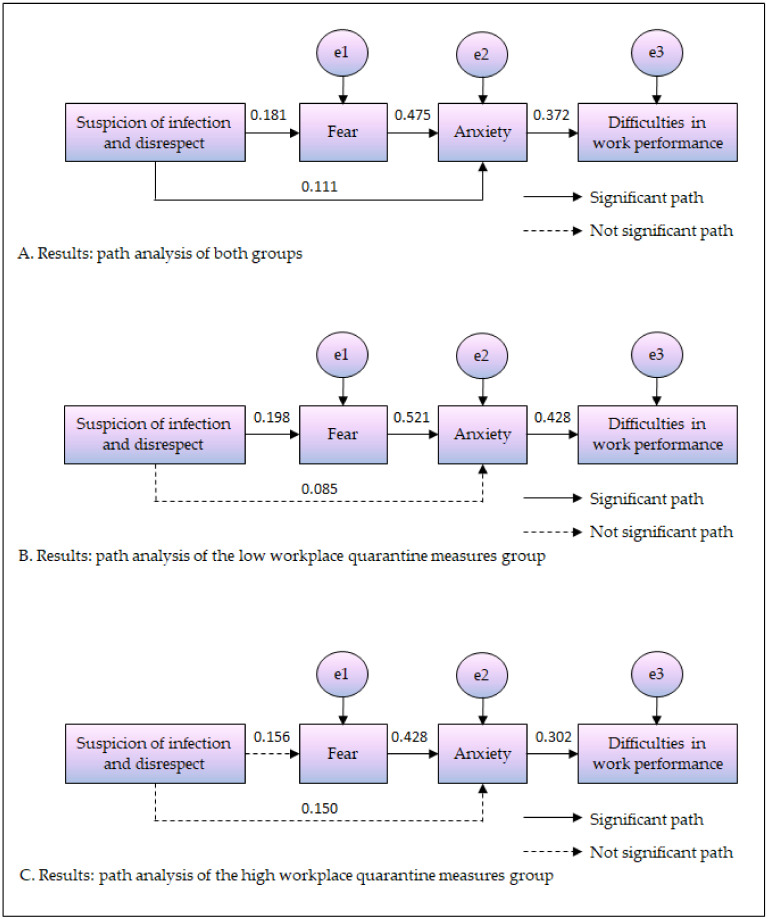
Result of path analysis. (**A**) Results: path analysis of both groups. (**B**) Results: path analysis of the low workplace quarantine measures qroup. (**C**) Results: path analysis of the high workplace quarantine measures group.

In the group with low workplace quarantine measures, anxiety had positive effects on difficulties in work performance (β = 0.428, *p* < 0.001). Moreover, in the group with high workplace quarantine measures, anxiety had positive effects on difficulties in work performance (β = 0.302, *p* = 0.001). In both groups, higher anxiety led to more difficulties in work performance. There was a significant difference in the path for anxiety and difficulties in work performance between the two groups (Critical Ratios = 2.742 > 1.960) (Table 4 and Figure 2).

## 4. Discussion

A hypothetical model was established to explain the difficulties in work performance of visiting nurses during the COVID-19 pandemic and to identify the influencing factors. This study analyzed the effects of workplace quarantine measures on experiences of verbal abuse (suspicion of infection and disrespect) and difficulties in work performance. To investigate the mediating effects of fear and anxiety, multi-group path analysis was conducted.

Our findings showed that more experiences of verbal abuse (suspicion of infection and disrespect) led to increased fear. Verbal abuse in hospitals causes mental problems, and most victims of verbal abuse experience fear and show emotional responses such as fearing to be with patients [20]. This is consistent with our finding that experiences of verbal abuse (suspicion of infection and disrespect) increase fear. In contrast, in groups with high workplace quarantine measures, experience of verbal abuse did not affect fear. This suggests that high levels of quarantine measures in workplaces prevents the experience of verbal abuse (suspicion of infection and disrespect) from affecting difficulties in work performance. In a study by Park [21], healthcare professionals working outside of medical institutions during the COVID-19 spread period showed evident symptoms of fear and anxiety. This was attributed to the low access to protective equipment and COVID-19 infection prevention methods compared with those in medical institutions [21]. In a study on Spanish nurses, the situation of workplace bullying affects internal satisfaction through social support [22]. This can be said that the violence experience affects the emotional state, and in the process, social support such as workplace quarantine affects. These findings suggest that the fear of visiting nurses may be reduced with increased quarantine measures in workplaces, such as the wearing of protective equipment and other strategies to prevent infection. Therefore, it is necessary to prepare policies to strengthen quarantine measures in workplaces to prevent difficulties in performing work for visiting nurses in preparation for possible new infectious disease pandemics in the future.

More experiences of verbal abuse (suspicion of infection and disrespect) were associated with greater anxiety. On the other hand, differences in the level of quarantine measures in workplaces showed that the experience of verbal abuse (suspicion of infection and disrespect) did not affect anxiety. This finding indicated that the level of quarantine measures in workplaces did not have effects on the path for experience of verbal abuse (suspicion of infection and disrespect) and anxiety. Therefore, experiences of verbal abuse must be curtailed to reduce anxiety and thereby prevent difficulties in work performance of visiting nurses. In a study on Korean workers [23], workplace violence, including verbal abuse, had a statistically significant relationship with anxiety. Anxiety was 4.1 times higher in those who experienced workplace violence, including verbal abuse, compared to those who did not, suggesting that intervention programs on the prevention of verbal and workplace violence need to be developed for the mental health of workers [23]. In clinical environments, nurses are greatly vulnerable to verbal abuse from patients and guardians [24]. Therefore, it is necessary to establish and introduce a system for the prevention of verbal abuse and the improvement of awareness by patients and guardians.

COVID-19-related fear led to increased anxiety. In addition, in both groups with high and low levels of workplace quarantine measures, fear had significant effects on anxiety. Im et al. [25] previously showed that fear of COVID-19 was a significant predictor of anxiety, and that greater fear of COVID-19 was associated with increased likelihood of being in an anxiety-risk group. In particular, women were more likely to be in the anxiety-risk group than men. In a study on the general public by Lee et al. [26], psychological difficulties and fear were variables that affected the probability of belonging to a high anxiety group. Similarly, Park et al. [27] reported that fear of social stigma caused by COVID-19 increased anxiety in adults. As such, our finding that COVID-19-related fear increases anxiety was in agreement with previous findings [25,26,27]. Therefore, mental health management systems that can detect mental health problems at an early stage, before anxiety is caused by fear of infectious disease pandemics, must be introduced. In addition, to alleviate emotional responses such as fear, strategies, including professional counselling, must be sought.

Greater anxiety was associated with increased difficulties in work performance. In addition, in both groups with high and low levels of workplace quarantine measures, anxiety had significant effects on difficulties in work performance. In previous studies on general hospital nurses [28,29], among the socio-psychological factors, lower anxiety led to significantly improved quality of nursing. Altogether, these findings support our study’s finding that higher anxiety leads to more difficulties in work performance. Therefore, intervention programs that reduce psychological and mental problems before high anxiety creates difficulties in work performance, must be sought, and effective management strategies devised.

In conclusion, in the path model of the group with high levels of workplace quarantine measures, experience of verbal abuse (suspicion of infection and disrespect) did not affect fear. This indicates that when the level of quarantine measures in workplaces is high, experiences of verbal abuse (suspicion of infection and disrespect) do not affect difficulties in work performance. In addition, the effects between variables were reduced in the group with high levels of workplace quarantine measures. These findings provide useful evidence for the increase in levels of quarantine measures in workplaces.

## 5. Limitations

Several limitations must be considered in interpreting the study’s findings. First, the participants were selected through convenience sampling for rapid collection of data during the COVID-19 pandemic. Therefore, the findings cannot be generalized to all visiting nurses. Second, data were collected by self-reporting using questionnaires. The self-report has a limitation in that it can include the subjective opinions of respondents. Third, quarantine, fear, and anxiety related to the possibility of spreading COVID-19 infection only considered the aspect of the workplace. I hope that follow-up studies will be conducted in the future, considering personal aspects such as individual quarantine, nurses’ families. However, this study is meaningful as it investigated the mediating effects of fear and anxiety in the relationship of experiences of verbal abuse (suspicion of infection and disrespect) that affect difficulties in work performance in visiting nurses during a pandemic of a new infectious disease. In addition, this study is significant as it showed that experiences of verbal abuse did not affect difficulties in work performance when the levels of quarantine measures at workplaces were high.

## 6. Conclusions

This multi-group path analysis study investigated the effects of verbal abuse (suspicion of infection and disrespect) on difficulties in work performance according to workplace quarantine measures in visiting nurses during the COVID-19 pandemic. In the total pathway model, experiences of verbal abuse mediated fear and anxiety to affect difficulties in work performance. In particular, in the path model of the group with a high level of workplace quarantine measures, experiences of verbal abuse (suspicion of infection and disrespect) did not directly affect fear. In contrast, in the group with a low level of workplace quarantine measures, experiences of verbal abuse (suspicion of infection and disrespect) had direct effects on fear.

The following are suggested based on the findings of this study: First, to prevent verbal abuse by patients and their guardians, a structured system in workplaces and preventive education for improved awareness must be provided. Second, in preparation for possible pandemics of new infectious diseases in the future, workplace quarantine policies must be established to prevent difficulties in work performance for visiting nurses. Third, mental health management systems within workplaces and intervention programs may be necessary to detect the early fear and anxiety of visiting nurses. These proposals will affect not only the life as a visiting nurse but also the quality of personal life.

## Figures and Tables

**Table 1:** Descriptive statistics of variables.

Variables	Range	M	SD	Skewness	Kurtosis
Suspicion of infection and disrespect	0–50	1.810	5.565	6.277	46.870
Ln (Suspicion of infection and disrespect +1)	0–3.93	0.489	0.829	1.733	2.664
Fear	18–40	33.637	5.297	−0.688	−0.209
Anxiety	1–10	7.940	1.851	−0.763	0.514
Difficulties in work performance	0–10	9.060	1.554	−1.987	5.067
Workplace quarantine measures	1–13	4.366	2.234	0.902	1.124

**Table 2:** Result of path analysis.

Path			B	SE	β	C.R.	*p*
Suspicion of infection and disrespect	→	Fear	10.154	0.389	0.181	20.969	0.003
Suspicion of infection and disrespect	→	Anxiety	0.247	0.121	0.111	20.041	0.041
Fear	→	Anxiety	0.166	0.019	0.475	80.753	<0.001
Anxiety	→	Difficulties in work performance	0.377	0.058	0.372	60.473	<0.001

**Table 3:** Indirect effect of independent variables on Difficulties in work performance.

Path	Indirect Effect	SE	95% CI		*p*
			LLCI	ULCI	
Suspicion of infection and disrespect→ Fear→Anxiety→Difficulties in work performance	0.081	0.028	0.034	0.143	0.001
Suspicion of infection and disrespect→ Anxiety→Difficulties in work performance	0.104	0.046	0.022	0.201	0.017

**Table 4:** Result of Path analysis according to Workplace quarantine measures.

Path			Workplace Quarantine Measures						Critical Ratios
			Low			High			
			β	C.R.	*p*	β	C.R.	*p*	
Suspicion of infection and disrespect	→	Fear	0.198	2.536	0.011	0.156	1.600	0.110	0.936
Suspicion of infection and disrespect	→	Anxiety	0.085	1.247	0.212	0.150	1.703	0.089	−0.456
Fear	→	Anxiety	0.521	7.649	<0.001	0.428	4.864	<0.001	2.785
Anxiety	→	Difficulties in work performance	0.428	5.946	<0.001	0.302	3.204	0.001	2.742

## Data Availability

Not applicable.

## References

[1] Cha K.S., Shin M.J., Lee J.Y., Chun H.K. (2017). The role of infection control nurse during emerging infectious disease epidemic: Focusing on the middle east respiratory syndrome. Korean J. Healthc.-Assoc. Infect. Control Prev..

[2] Central Disease Control Headquarters (2021). Cases in Korea (Internet). http://ncov.mohw.go.kr/.

[3] Choi I.H., Chung Y.H., Park I.H., Choi Y.A. (2013). Job stress, Organizational commitment, way of coping and turnover intention among Korean visiting nurses. Korean J. Occup. Health Nurs..

[4] Jun S.H., Lee M.H., Choi M.J. (2021). COVID-19 Infection Control-Related Fatigue, Job Stress, and Burnout in Nurses. J. Korean Acad. Soc. Home Care Nurs..

[5] Holroyd E., McNaught C. (2008). The SARS crisis: Reflections of Hong Kong nurses. Int. Nurs. Rev..

[6] Kyung D.E., Shin Y.S. (2021). Factors Associated with Nurses’ Nursing Intention toward COVID-19 Patients. Korean J. Adult Nurs..

[7] Jin D., Lee G. (2020). Experiences of nurses at a general hospital in Seoul which is temporarily closed due to COVID-19. J. Korean Acad. Soc. Nurs. Educ..

[8] Giusti E.M., Pedroli E., D’Aniello G.E., Badiale C.S., Pietrabissa G., Manna C., Badiale M.S., Riva G., Castelnuovo G., Molinari E. (2020). The psychological impact of the COVID-19 outbreaks on health professionals: A cross-sectional study. Front. Psychol..

[9] Hu D., Kong Y., Li W., Han Q., Zhang X., Zhu L.X., Wan S.W., Liu Z., Shen Q., Yang J. (2020). Frontline nurses’ burnout, anxiety, depression, and fear statuses and their associated factors during the COVID-19 outbreak in Wuhan, China: A large-scale cross-sectional study. EClinicalMedicine.

[10] Liu Q., Luo D., Haase J.E., Guo Q., Wang X.Q., Liu S., Xia L., Liu Z., Yang J., Yang B.X. (2020). The experiences of health-care providers during the COVID-19 crisis in China: A qualitative study. Lancet Glob. Health.

[11] Canton A.N., Sherman M.F., Magda L.A., Westra L.J., Pearson J.M., Raveis V.H., Gershon R.R.M. (2009). Violence, job satisfaction, and employment intentions among home healthcare registered nurses. Home Healthc. Now.

[12] Lee I.S., Lee K.O., Kang H.S., Park Y.H. (2012). Violent Experiences and Coping among Home Visiting Health Care Workers in Korea. J. Korean Acad. Nurs..

[13] Hwang Y.Y., Park Y., Park S. (2015). Experience of workplace violence among intensive care unit nurses. Korean J. Adult Nurs..

[14] Lee M.H., Kim M.Y., Go Y.J., Kim D.R., Lim H.N., Lee K.H., Yang S.Y. (2021). Factors Influencing in the Infection Control Performance of COVID-19 in Nurses. J. Digit. Converg..

[15] Bae J.Y., Lee E.K., Kim B.J., Lee E.J. (2021). The Influencing Factors of Burnout in Nurses in the COVID-19 Pandemic Disaster. Korean J. Stress Res..

[16] Bae M.Y., Jeong J.Y. (2021). The Effect of Perceived Risk of COVID-19 and Quarantine Levels on Seoul Destination Image, Attitude, and Behavioral Intentions: Focused on Mediating Effects of Quarantine Level. J. Tour. Stud..

[17] Kim J.H., Oh J.H., Kim T.Y., Yoo J.G., Choi H.J., Lee D.G., Kim J.S., Park W.I. (2020). COVID-19 Issue & Diagnosis from Gyeonggi-do Residents’ Perspective.

[18] Hair J.F., Black W.C., Babin B.J., Anderson R.E., Tatham R.L. (2006). Multivariate Data Analysis.

[19] Bea B.R. (2014). Structural Equation Modeling with LISREL 9.1: Principles and Practice.

[20] Arnetz J.E., Arnetz B.B. (2001). Violence towards health care staff and possible effects on the quality of patient care. Soc. Sci. Med..

[21] Park S.M. (2020). The impact of the COVID-19 pandemic on mental health among population. Korean J. Health Educ. Promot..

[22] del Carmen Pérez-Fuentes M., Gázquez J.J., del Mar Molero M., Oropesa N.F., Martos Á. (2020). Violence and job satisfaction of nurses: Importance of a support network in healthcare. Eur. J. Psychol. Appl. Leg. Context.

[23] Choi E.S., Jung H.S., Kim S.H., Park H. (2010). The Influence of Workplace Violence on Work-related Anxiety and Depression Experience among Korean Employees. J. Korean Acad. Nurs..

[24] Jeong A.J., Jang K.A. (2020). Effects of Post-Traumatic Stress, Tissue Immersion and Job Satisfaction by Linguistic Violence Experiences. J. Korean Soc. Oral Health Sci. Vol..

[25] Lim H.B., Lee D.E., Choi Y.K., Lee J.S. (2021). The Effect of Fear of COVID-19, Perceived Risk of COVID-19 and Resilience on Anxiety. Korean J. Psychol..

[26] Lee D.H., Kim Y.J., Lee D.H., Hwang H.H., Nam S.K., Kim J.H. (2020). The Influence of Public Fear, and Psycho-social Experiences during the Coronavirus Disease 2019(COVID-19) Pandemic on Depression and Anxiety in South Korea. Korean J. Psychol..

[27] Park H.J., Kim M.S., Kim S.H., Song G.R. (2021). The Relationship Between Fear of Social Stigma According to Adult’s COVID-19 Infection, Change in Quality of Life, and COVID-19 Anxiety: The Mediating Effects of Perceived Situational Control. J. Korean Assoc. Psychother..

[28] Lee M.J., Yoon S.H., Cho Y.C. (2016). Relationship between Psychosocial Factors, Job Stress Contents, Fatigue Symptoms and Quality of Nursing Services among General Hospital Nurses. J. Korea Acad.-Ind. Coop. Soc..

[29] Lee M.J., Cho Y.C. (2015). Analysis of the Influencing Factors on Quality of Nursing Services in General Hospital Nurses using the Structural Equation Model. J. Korea Acad.-Ind. Coop. Soc..

